# Evaluating Aromaticity in Ag_6_Ru and Ag_10_Ru as Electron‐Precise Superatom Clusters

**DOI:** 10.1002/cphc.202401118

**Published:** 2025-04-17

**Authors:** Mesías Orozco‐Ic, Peter L. Rodríguez‐Kessler, Alvaro Muñoz‐Castro

**Affiliations:** ^1^ Instituto de Ciencias Físicas Universidad Nacional Autónoma de México Cuernavaca 62210 México; ^2^ Centro de Investigaciones en Óptica A.C. Loma del Bosque 115, Col. Lomas del Campestre León Guanajuato 37150 Mexico; ^3^ Facultad de Ingeniería Arquitectura y Diseño Universidad San Sebastián Bellavista 7 Santiago 8420524 Chile

**Keywords:** aromaticity, clusters, current‐density, magnetic response, ruthenium, silver

## Abstract

Herein, the superatomic characteristics of the favorable global minima of electron precise clusters are evaluated, leading to stable species featuring catalytic reactive sites and inherent aromaticity in both planar and spherical realms. The results show that Ag_6_Ru exhibits an electron‐precise ten‐electron 1S^2^1P
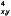
1D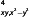 planar superatomic electron shell structure, related to the 1S^2^1P^6^1D^10^ 18‐electron structure of the spherical Ag_10_Ru cluster, involving seven and ten reactive sites. The favorable electronic structure in such related clusters exhibits diatropic ring currents and long‐range shielded regions, supporting the respective planar‐ and 3D aromatic character. Hence, further planar and 3D relationship between electron precise clusters may trigger a fundamental rationalization in finding stable and useful targets for undergoing catalytic activity for reactions such as oxygen reduction reaction, extending similarities between different cluster shapes at certain sizes.

## Graphical abstract

An exhaustive study of Ag_6_Ru and Ag_10_Ru clusters reveals their stability and aromaticity. The analysis shows these species possess precise electron shell structures, classifying them as planar and spherical superatoms. Ag_6_Ru exhibits a ten‐electron shell, while Ag_10_Ru shows 18 electrons. Both display strong diamagnetic responses and support diatropic ring currents, confirming aromatic character.

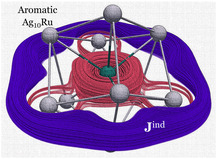

## 1 Introduction

In recent years, there has been a significant increase in research on silver clusters, driven by their distinctive stability and reactivity. Extensive studies on both doped silver clusters and silver alloys have yielded valuable insights into their properties and potential applications in areas such as optics, sensors, and catalysis.^[^ ^1^, ^2^, ^3^, ^4^, ^5^ ^]^ Numerous theoretical and experimental studies have been dedicated to exploring the structure–performance relationships of both charged and neutral silver clusters, with particular stability provided by electronic shell closure.^[^ ^6^, ^7^ ^]^ The size‐dependent properties of these clusters, driven by quantum size effects, can be strategically manipulated to optimize their persistence for specific nuclearity, enabling further applications. Particularly, their optical characteristics are well‐suited for plasmonics, where they can amplify electromagnetic fields at the nanoscale, thereby advancing the development of sensing and photonic devices.^[^ ^8^ ^]^ A deep understanding of the structure and stability of silver clusters is essential for effectively leveraging their unique properties. Recent advances in both computational and experimental techniques have enabled researchers to examine the atomic arrangements and electronic structures of these clusters with remarkable precision. These methodologies encourage the detailed understanding of the geometric configurations, electronic states, and dynamic behaviors of silver clusters under different conditions. In literature, a large number of theoretical and experimental works have been devoted to understanding the effects and modifications in the structural and electronic properties of clusters doped with early 3d transition metals, as well as noble metals.^[^ ^9^, ^10^ ^]^ Most of these studies showed that the clusters show quenching of the magnetic moments at specific sizes.^[^ ^5^, ^11^, ^12^, ^13^, ^14^, ^15^ ^]^ For instance, interesting modifications in the structure of doped‐Ag clusters have been found in Ag_6_M, Ag_9_M and Ag_10_M (M = Co, Rh, Ru), in comparison with the bare Ag_*n*_ counterpart.

On the other hand, aromaticity plays an important role in explaining the existence and stability of both planar and nonplanar organic and inorganic molecules and increasing its use in recent years.^[^ ^16^, ^17^, ^18^ ^]^ However, understanding the stability of superatomic clusters from the aromaticity point of view has been barely explored.^[^ ^19^, ^20^, ^21^ ^]^ It is important to note that determining whether a molecule is aromatic or not is not a trivial task, particularly for systems containing heavy elements. However, calculating the molecular magnetic response to an external magnetic field is a promising approach to quantifying aromaticity. This analysis must be performed with caution due to the contribution of core electrons, which are unrelated to the aromaticity itself.^[^ ^22^, ^23^ ^]^

In order to get insight into the bonding and aromaticity properties of Ag_*n*_M clusters, we set to account for the electronic, optical, and magnetic properties related to the formation of electron‐precise Ag_6_Ru and Ag_10_Ru clusters. Such species enables evaluation of planar and spherical species following the planar and spherical electronic shell closure for superatoms,^[^ ^24^ ^]^ favoring the understanding under two different cluster arrays. Spherical superatoms follow the consecutive electronic shell structure provided by 1S^2^1P^6^1D^10^ …, which is fulfilled by Ag_10_Ru, by the ten 5s^1^ electrons from each Ag center, and eight by the 4d^8^ atomic shell from the inner Ru atom. In contrast, the planar superatoms, with lack of the z‐axis, fulfil a related electronic shell closure, now on a 2D potential well, leading to 1S^2^1P
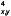
1D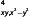, accounted by the six Ag and the central Ru atom. Such understanding favors comparison and understanding in both planar and spherical structural scenarios, of the cluster properties, which can be extended to different transition metal clusters.

## 2 Computational Details

Calculations performed in this work are carried out using density functional theory (DFT) as implemented in the Orca quantum chemistry package version 6.0.0.^[^ ^25^ ^]^ The exchange and correlation energies are addressed by the PBE0 functional in conjunction with the Def2TZVP basis set.^[^ ^26^, ^27^ ^]^ Atomic positions are self‐consistently relaxed through a quasi‐Newton method employing the Broyden–Fletcher–Goldfarb–Shanno (BFGS) algorithm. The Self‐Consistent field convergence criteria for geometry optimizations are achieved when the total energy difference is smaller than 10^−8^ au, using the TightSCF keyword in the input. The Van der Waals interactions are included in the exchange‐correlation functionals with empirical dispersion corrections of Grimme DFT‐D3 (BJ).

Vibrational frequencies were computed within the harmonic approximation, with the Hessian matrix obtained numerically. While this approach provides a reliable first‐order description, low‐frequency modes may exhibit anharmonic effects due to shallow potential energy surfaces. Nonetheless, the harmonic treatment remains a standard and effective method for theoretical–experimental comparisons in metal clusters. The total density of states (DOS) and partial density of states (PDOS) for clusters and complexes were obtained using the Multiwfn program.^[^ ^28^ ^]^ To assess the aromaticity for Ag_6_Ru and Ag_10_Ru clusters, we computed the magnetically induced current density^[^ ^29^, ^30^, ^31^ ^]^ ($J^{\text{ind}}$) and the induced magnetic field^[^ ^32^, ^33^, ^34^ ^]^ ($B^{\text{ind}}$) using the gauge‐including magnetically induced currents (GIMIC)^[^ ^29^, ^30^, ^31^ ^]^ and Aromagnetic^[^ ^35^ ^]^ programs, respectively. These calculations employed gauge‐including atomic orbitals^[^ ^36^ ^]^ within the Gaussian 16 software. The BHandHLYP functional was chosen for both $J^{\text{ind}}$ and $B^{\text{ind}}$ calculations due to its accuracy in reproducing magnetic properties comparable to those obtained at the coupled cluster singles doubles perturbative triples (CCSD(T)) level.^[^ ^37^ ^]^ An external magnetic field of 1T‐strength, aligned with the *z*‐axis (the axis of highest molecular symmetry), was applied. For planar molecules, the field was oriented perpendicular to the molecular plane. Under these conditions, the *z*‐component of $B^{\text{ind}}$ ($B_{z}^{\text{ind}}$) is typically the most significant, allowing us to focus on $B_{z}^{\text{ind}}$, which corresponds to the *zz*‐component of the nucleus‐independent chemical shift (NICS_*zz*_).^[^ ^38^, ^39^, ^40^ ^]^ Ring‐current strengths are reported in nA T^−1^, while $B_{z}^{\text{ind}}$ is given in ppm.

## 3 Results

**Figure 1:**
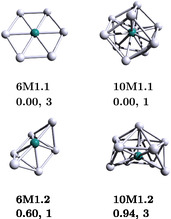
Lowest energy structures for Ag_6_Ru and Ag_10_Ru clusters are presented. For each structure, the relative energy (in eV) and corresponding isomer label are provided. Adapted with permission.^[^ ^41^ ^]^

The structural evolution and stability of Ag_*n*_Ru (*n* = 3–10) clusters have been systematically investigated in previous studies using genetic algorithms as implemented in the CLUSTER 1.0 code.^[^ ^41^, ^42^ ^]^ In order to study the bonding and aromaticity properties of Ag_6_Ru and Ag_10_Ru clusters, we reoptimized their ground‐state structures, as depicted in **Figure** **1**. The clusters are labeled by the **xM1.y** notation, in which **x** denotes the number of Ag atoms, and **y** indicates the isomer number. Interestingly, in these sizes we found that the most stable clusters (**6M1.1**, **10M1.1**) are significantly lower in energy compared to their low‐lying isomers (**6M1.2**, **10M1.2**), with 0.60 and 0.94 eV, respectively. The average Ru—Ag bond distance of **6M1.1** amounts to 2.72 Å, which is similar to the Ag–Ag ones, according to the D_6_ *_h_* symmetry. For **10M1.1** the average Ru—Ag bond distances amount to 2.66 Å, which are smaller larger than those of Ag–Ag, with 2.95 Å, which helps to characterize the structures of the clusters.

**Figure 2:**
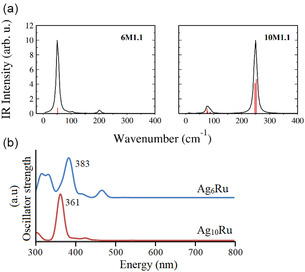
a) IR spectra and b) UV–vis absorption patterns for **6M1.1** and **10M2.1** clusters obtained at the PBE0/Def2TZVP level.

To gain insight into structural identification, we have also calculated the infrared (IR) spectra of the two most stable clusters. The characteristic peaks for **6M.1** and **10M1.1** clusters were found at 51.64, and 252.76 cm^−1^, suggesting remarkable differences in their vibrational modes. The IR spectra of the clusters show significant dispersion for **10M1.1**, while it is more localized for **6M.1** (**Figure** **2**). The lowest vibrational frequencies for **6M1.1** and **10M1.1** clusters are found at 18.12, and 10.61 cm^−1^, while the highest vibrational frequencies are at 202.09, and 252.76 cm^−1^, indicating a narrow range of their IR spectra. Despite the high symmetry of the Ag_6_Ru cluster, which would typically lead to IR‐inactive modes, our calculations reveal notable IR signals. This apparent contradiction arises from symmetry‐breaking effects in vibrational modes. Specifically, asymmetric stretching and out‐of‐plane vibrations induce temporary dipole moment variations, allowing IR absorption.

Additionally, theoretical studies have demonstrated that simulated IR and Raman spectra can be valuable tools for the structural identification of metal clusters, as previously reported for silver‐based systems.^[^ ^43^ ^]^ Furthermore, to complement the vibrational analysis and provide additional spectroscopic fingerprints, we computed the UV–vis absorption spectra of the studied clusters (Figure 2b). The main absorption features appear at 383 nm for **6M.1** and 361 nm for **10M1.1**, indicating significant differences in their optical properties.

These results align with previous studies where optical absorption has been used to identify the most stable structural isomers of silver‐based clusters. The agreement between our calculated spectra and experimental trends in similar systems further supports the robustness of our theoretical approach. These findings can serve as a contrast for future experiments when they become available.

**Figure 3:**
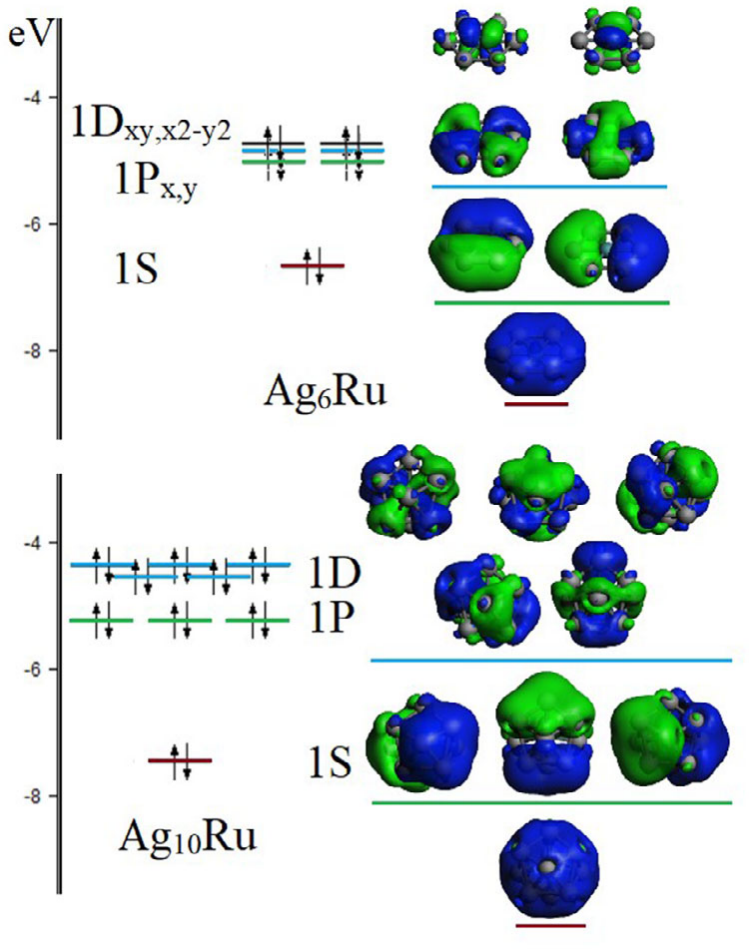
Electronic structure for Ag_6_Ru and Ag_10_Ru, denoting superatomic shells. Color code: 1S, red; 1P, green; 1D, blue.

The related ionization potential (IP) and electron affinity (EA) parameters of the clusters were valuated using the PBE0‐Def2TZVP level, while their formulas and definitions can be revised elsewhere. For Ag_6_Ru, Ag_10_Ru, clusters, the IP parameter amounts to 8.53 and 6.23 eV, while the EA amounts to 1.99 and 1.25 eV, respectively. The stability of the clusters is further analyzed through the chemical hardness (*η*) and chemical potential (*μ*), which can be derived from the IP and EA values. Both *η* and *μ* show similar trends compared to IP and EA but with more pronounced size‐dependent variations.^[^ ^41^ ^]^ These results motivate us to further examinate the superatomic and aromatic characteristics of the clusters. Their superatomic shell structures are given in **Figure** **3**. For the planar Ag_6_Ru structure, shells resembling zero, one, and two angular nodes are found, which follow the planar superatomic model^[^ ^24^, ^44^ ^]^ derived by the combination of six 5s‐Ag and the central 5d‐Ru atomic shells. Under a planar potential well, the resulting shells avoid the *z*‐axis, leading to 1S, 1P_*x*,*y*_, and 1D_*xy*,*x*2*−y*2_ superatomic shells fulfilling a closed shell situation for 2, 2 + 4, and 2 + 4 + 4 electron counts. In this sense, it features an electron‐precise planar superatomic species for an electron count of 10 in an electronic shell structure given by 1S^2^1P
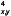
1D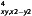, with the addition of a set of two 5d–Ru lone pairs as part of the frontier orbitals. Such a situation suggests a favorable electronic structure able to sustain a planar aromatic behavior in an all‐metal cluster, which is in line with the 3D superatomic clusters involving a spherical aromatic behavior.^[^ ^45^, ^46^, ^47^ ^]^ Next, Ag_10_Ru, having a 3D shape, involves a filled 1S^2^1P^6^1D^10^ 18‐electron structure, ascribed as a superatomic cluster, which is suggested to enable a spherical aromatic character. Hence, the comparison between Ag_6_Ru and Ag_10_Ru clusters favors the understanding of related planar‐ and 3D superatomic clusters that are likely to feature aromatic characteristics, thus favoring the correlation between clusters in different shapes. Interestingly, both species show an electronic structure filled up to two‐node shells ascribed as 1S 1P 1D, in the respective planar‐ and 3D realms.

**Figure 4:**
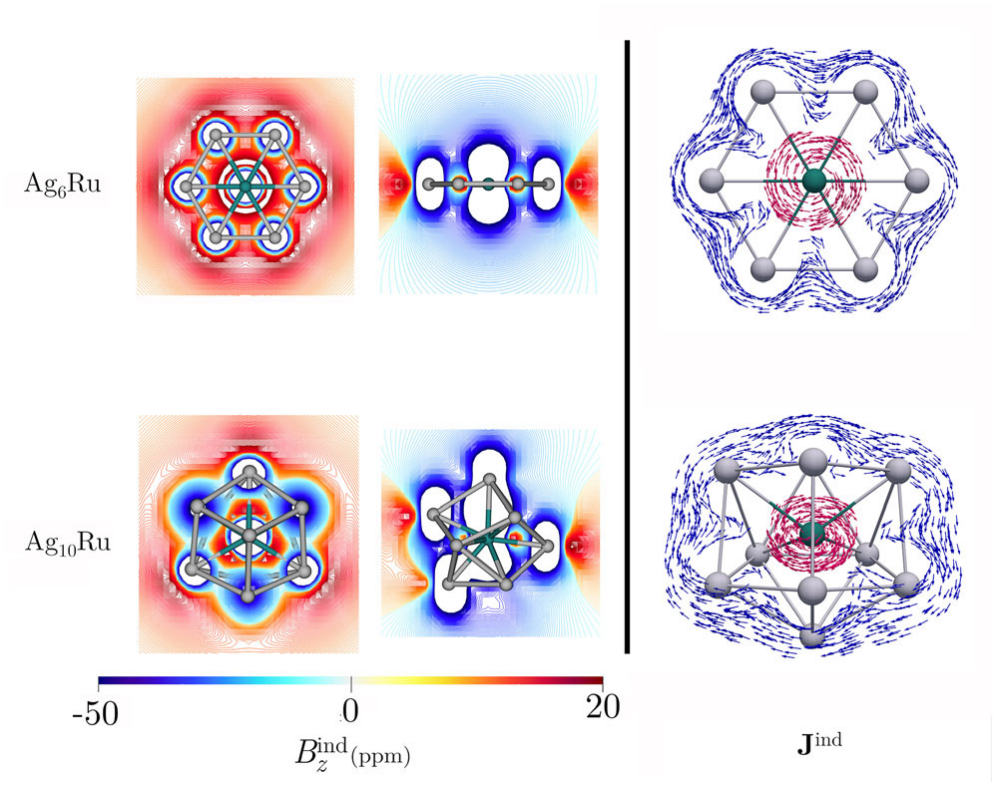
In the left panel: $B_{z}^{\text{ind}}$ isolines plotted in the molecular plane (left) and a transversal slide (right). In the right panel: $J^{\text{ind}}$ vectors plotted near the molecular plane/surface. $J^{\text{ind}}$ arrows in blue indicate the diatropic flux following the ring current pathways, while the magenta loops indicate the flux of the core–electron currents. These calculations were performed at the BHandHLYP/def2‐TZVP level.

Alternatively, it is possible to relate the stability of the systems in terms of aromaticity.^[^ ^47^, ^48^ ^]^ Thus, the aromaticity features of Ag_6_Ru and Ag_10_Ru clusters were addressed via the analysis of the magnetic response by calculating the magnetically induced current density^[^ ^29^, ^30^, ^31^ ^]^ ($J^{\text{ind}}$) and the induced magnetic field ($B^{\text{ind}}$).^[^ ^32^, ^33^, ^34^ ^]^ Both the planar and 3D cases exhibit a strongly diamagnetic response, which is characterized by a shielding cone with long‐range $B_{z}^{\text{ind}}$ negative values over the entire molecular structure (**Figure** **4**), which is typical of aromatic systems. However, describing aromaticity in terms of the shielding function alone is not sufficient when heavy elements are involved due to the strong contributions from core electrons.^[^ ^22^, ^23^ ^]^ In that sense, $J^{\text{ind}}$ calculations are more useful, as they allow us to confirm that indeed both systems support a diatropic (clockwise flowing) ring current such as the case of benzene.^[^ ^29^, ^30^, ^31^ ^]^ For the case of Ag_6_Ru the current density flows along the six‐membered silver ring while for Ag_10_Ru, the diatropic flux appears outside the Ag_10_ cage‐like surface (Figure 4). The calculation of the ring‐current strength by the $J^{\text{ind}}$ integration across a plane intersecting one or more chemical bonds is promising for quantitative comparison of aromaticity. However, the presence of Ru atoms in the center complicates the correct estimation,^[^ ^22^, ^23^ ^]^ so we have circumvented the core‐electron flux (see Figure 4) from it in our integration, leading to a ring‐current strength of 8.27 and 20.40 nA T^−1^, respectively, for Ag_6_Ru and Ag_10_Ru. This confirms a remarkable aromatic character for these systems.

**Figure 5:**
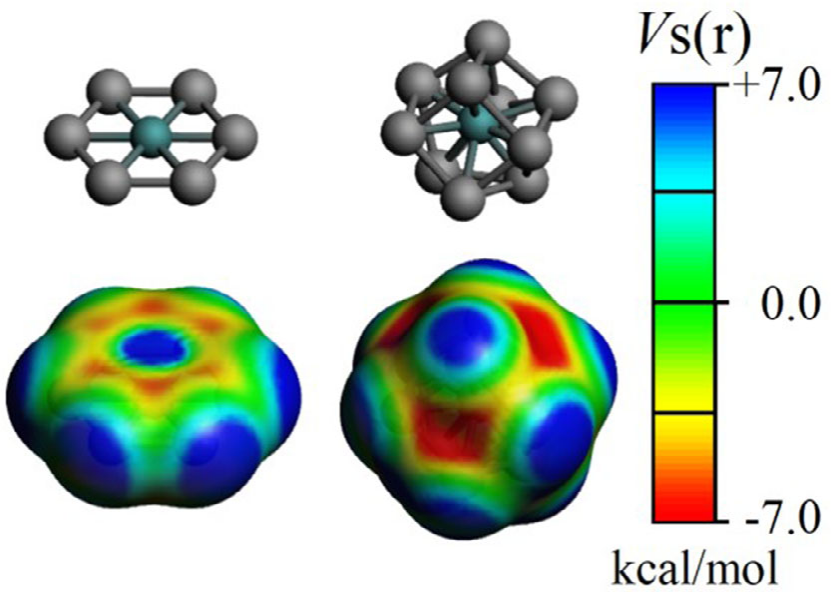
Molecular electrostatic potential (Vs(r)) over the van der Waals surface for Ag_6_Ru (left) and Ag_10_Ru (right). Holes noted as most positive regions (blue).

Silver clusters have been depicted as active species for oxygen reduction reaction (ORR),^[^ ^49^ ^]^ promoting the evaluation of plausible reactive sites in such planar‐ and 3D superatomic silver‐doped clusters. From the molecular electrostatic potential (Vs(r)), such sites can be effectively located^[^ ^50^ ^]^ as the formation of local holes over the van der Waals surface with positive potential regions. For Ag_6_Ru and Ag_10_Ru clusters, the reactive sites noted as the formation of holes over the overall structure as maximum values of the electrostatic potential (Vs(r), max) in **Figure** **5** reveal active sites at the contour of the Ag_6_ border and Ag_10_ cage. Thus, such species offer stabilization of six and ten favorable regions for undergoing catalytic activity in planar‐ and 3D structural aggregates as prospects for ORR capabilities, which can be controlled by size selection. In addition, for Ag_6_Ru, the supported and accessible Ru atom introduces a seventh site, increasing the reactive versatility in such species. This observation appears relevant in determining plausible cluster sizes that are useful for application in the ORR activity, driven by the reduction of the border site effect on the cluster global minima.^[^ ^50^ ^]^ Hence, both related species are relevant small‐sized clusters sharing stable superatomic features with electron precise counts, which may encourage further finding and evaluation of planar‐ and 3D shapes of related doped clusters, which can be tailored by modifying their chemical composition.^[^ ^51^ ^]^

## 4 Conclusion

We have unraveled a relationship between planar‐ and 3D cluster shapes of favorable species, driven by their electron precise electronic structures of Ag_6_Ru and Ag_10_Ru. The rationalization of such clusters as planar and 3D superatoms featuring a ten‐electron 1S^2^1P
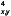
1D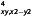 shell structure for Ag_6_Ru, and eighteen electron 1S^2^1P^6^1D^10^, favors finding of specific sizes with particular stability in different shapes, retaining seven and ten catalytic active sites, respectively. In addition, the superatomic character exhibits an inherent aromatic behavior confirmed via computations of the magnetic response, explaining their high stability. Both planar and 3D clusters sustain strong ring currents and shieldings. Hence, such species encourage further findings of planar and 3D relatives connected by electron‐precise electronic structures in different architectures and strong aromaticity, triggering the quest for extending similarities between different cluster shapes at specific sizes, sustaining catalytic active sites for reactions such as ORR.^[^ ^52^ ^]^

## Conflict of Interest

The authors declare no conflict of interest.
